# Immune Responses Against Allergic Asthma Following Intervention with *Lacticaseibacillus paracasei* DMLA16017 and Vitamin D in Rats

**DOI:** 10.3390/nu18111736

**Published:** 2026-05-28

**Authors:** Ao Xie, Lu Yuan, Biao Yang

**Affiliations:** 1 Department of Pathogen Biology and Microecology, College of Basic Medical Science, Dalian Medical University, Dalian 116044, China; wst_xie@dmu.edu.cn (A.X.); 15255715375@163.com (L.Y.); 2 Department of Pathogen Biology, School of Basic Medicine, Shenyang Medical College, Shenyang 110034, China

**Keywords:** DMLA16017, vitamin D, gut microbiome, allergic asthma, rat, immune response

## Abstract

Objectives: Allergic asthma (AA) is an increasing public health concern. The aim of this study was to investigate the potential effects of immune responses against AA in rats following intervention with *Lacticaseibacillus paracasei* DMLA16017 and vitamin D (VD). Methods: *L. paracasei* DMLA16017 was identified using 16S rDNA sequencing, while a rat model of AA was established via ovalbumin (OVA) induction. Subsequently, samples were collected for biomarker analysis in peripheral blood and lung tissue (including serum OVA-immunoglobulin E (IgE) and cytokines) using enzyme-linked immunosorbent assays and assessment of the composition of the intestinal microbiota and species diversity using 16S rRNA sequencing. Results: In the rat model, OVA-induced sensitization induced significant physiological alterations, including pulmonary tissue damage, elevated white cell counts, increased serum levels of OVA-IgE and cytokines interleukin (IL)-4 and IL-17, and reduced levels of IFN-γ and TGF-β. These changes were accompanied by dysbiosis of the gut microbiota and decreased species diversity. Co-administration of VD and DMLA16017 effectively ameliorated the physiological disturbances and histopathological abnormalities in rats with AA, restored the balance between cellular and immune responses, and improved the composition of the gut microbiota and species diversity. Conclusions: Combined intervention with VD and DMLA16017 can be used to treat AA disorders, with potential long-term modulation of the immune system.

## 1. Introduction

Asthma is a prevalent chronic respiratory disorder, and allergic asthma (AA) represents the most common phenotype. Key pathological features include airway hyperresponsiveness, chronic airway inflammation, bronchoconstriction, and excessive production of mucus, which lead to impaired pulmonary function and clinical symptoms such as wheezing, dyspnea, persistent cough, and respiratory distress [1]. Epidemiological studies indicate that over 300 million individuals suffer from allergic diseases, which poses a challenge to public health and results in considerable socioeconomic burden [1,2]. Therefore, comprehensive knowledge of the pathogenic mechanisms underlying AA, the identification of potential therapeutic targets, and the implementation of innovative preventive and interventional strategies are of paramount scientific importance and urgent practical necessity.

The pathogenesis of AA is highly complex, and the most prevalent trigger is prolonged exposure to allergens [3]. The imbalance between T helper 1 (Th1) and T helper 2 (Th2) cells constitutes a central mechanism that underlies hypersensitivity reactions in AA, which are specifically marked by the suppression of Th1 activity and predominant activation of Th2 [4]. Furthermore, the intestinal microbiota plays a pivotal role in the maintenance of immune homeostasis. Disruptions in the composition of the microbiota and immunoregulation are closely associated with the onset and progression of allergic disorders [5].

The composition of the microbial community is established during early life, and it remains subject to dynamic evolution throughout development. The initial colonization of microorganisms at mucosal sites proceeds in parallel with maturation of the immune system [6]. A hallmark of immune immaturity is the low production of cytokines, which results in attenuated inflammatory responses and creates a permissive environment for microbial colonization and proliferation across diverse ecological niches [7]. The gut microbiota can modulate the abundance of short-chain fatty acid (SCFA)-producing bacteria and fine-tune inflammatory pathways to exert significant effects on the health of the host.

Vitamin D (VD) comprises a group of fat-soluble steroid derivatives within the vitamin family and plays essential roles in the maintenance of skeletal integrity and homeostasis of calcium and phosphorus metabolism [8]. Growing attention has been directed toward synergistic interactions between the gut microbiome and VD in the regulation of host health and pathogenesis. Vitamin D deficiency can alter the composition of the gut microbiota and impair the structural and functional integrity of the intestinal epithelial barrier, which significantly affect the host’s microbial ecological balance [9]. Consequently, it is critical to maintain the level of VD within the normal physiological range to preserve homeostasis of the intestine.

There is a growing realization that interconnections between probiotics and the development of AA can facilitate the acquisition of hallmark capabilities. This study established an ovalbumin (OVA)-induced model of AA to evaluate the therapeutic efficacy of combined treatment using VD and *Lacticaseibacillus paracasei* DMLA16017. Furthermore, it investigated the correlation and scientific significance of cytokine profiles and the gut microbiota. The findings of this study provide a more comprehensive understanding of the therapeutic effect of combined intervention using VD and *L. paracasei* DMLA16017 and present a theoretical foundation for developing novel interventions against AA.

## 2. Materials and Methods

### 2.1. The Growth Curve and Identification of DMLA16017

DMLA16017 was cultured in MRS broth (Hopebio, Qingdao, China) at pH 5.7 ± 0.2 under strictly anaerobic conditions. Monoclonal colonies were uniformly spread onto glass slides, and then Gram staining was performed. The isolated and purified strain DMLA16017 was submitted to Sangon Biotech (Shanghai) Co., Ltd. (Shanghai, China) for 16S rDNA sequence analysis and identification. For growth curve analysis, DMLA16017 was inoculated into 45 mL of MRS liquid medium at an inoculum concentration of 2% and statically incubated at 37 °C. The optical density at 620 nm (OD620) was measured at predetermined time points: 0, 12, 24, 36, and 48 h post-inoculation. The growth curve was constructed with cultivation time on the *x*-axis and OD620 values on the *y*-axis. Concurrently, biomass changes were assessed using the plate colony counting method. Bacteria were harvested during the early stationary phase and stored at −80 °C.

### 2.2. Animal Model and Intervention Administration

Specific pathogen-free (SPF) male Wistar rats were used at 4~5 weeks of age (55 ± 3.22 g) and acclimatized for two weeks in the Animal Center of Dalian Medical University. The rats were housed under controlled environmental conditions (22 ± 2 °C, 50–60% relative humidity) with a 12 h light/dark cycle and free access to sterile food and sterile water.

**Figure 1:**
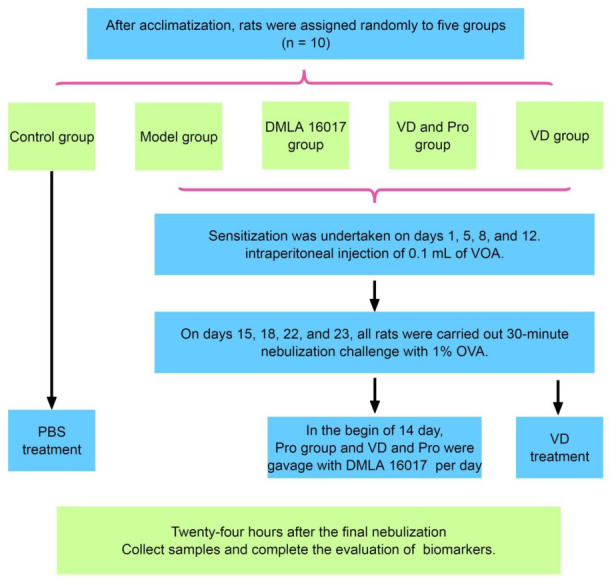
Experimental design of a model of AA in rats.

Based on the previously described improvement in airway inflammation, an AA model was established [10]. Following a 7-day acclimatization period, the rats were randomly assigned to five experimental groups (n = 10): the control group (Con), the model group (Model), the DMLA16017 group (Pro), the VD group (VD), and the VD combined with DMLA16017 group (VD and Pro). Sensitization was performed on days 1, 5, 8, and 12 in all groups except the Con group. The rats were sensitized to OVA (Sigma-Aldrich, Saint Louis, MO, USA) via intraperitoneal injection of 0.1 mL of alum-precipitated antigen, containing 75 μg OVA and 2 mg Al(OH)_3_ adjuvant. On days 15, 18, 22, and 23, all rats underwent a 30 min nebulization challenge with 1% OVA using an ultrasonic nebulizer (YuWell Medical Equipment & Supply Co., Ltd., Zhenjiang, China). After developing airway inflammation, the Pro group and the VD and Pro group were gavaged with 1.0 × 10^9^ colony forming units (CFU) of DMLA16017 suspended in 0.2 mL of PBS per day, 1 h before the nebulization challenge. Meanwhile, the VD group was gavaged with the 0.2 mL of VD, and the other groups were treated with PBS. All animals were humanely euthanized 24 h after the final nebulization. The rats were rapidly rendered unconscious by gradually increasing the concentration of carbon dioxide, avoiding the discomfort caused by a sudden rise in concentration. The gas flow rate was strictly controlled to achieve a final CO_2_ concentration of 30%, ensuring that only one animal was processed at one time. For detailed operational procedures, please refer to Figure 1.

To minimize pain, distress, and suffering during sensitization, OVA nebulization, and repeated gavage, the following measures were taken. Pain assessment was conducted to inform the development and implementation of a comprehensive pain management and welfare monitoring plan. Repeated oral gavage was performed exclusively by personnel certified in advanced rodent handling techniques, using appropriately sized, lubricated stainless-steel gavage needles; each procedure was executed with deliberate, controlled motion to avoid esophageal or gastric trauma and to minimize mechanical stress. To prevent additional stress to both the test subjects and other animals, sensitization and OVA nebulization were conducted in a calm environment and carried out in areas where other animals could not see or smell the procedure. Ten rats were included in each group for model construction. All animals completed the entire experimental intervention, and finally, six model rats were randomly selected to collect experimental data for the final analysis. All animals were euthanized at the end of the experiment, and no premature deaths occurred during the course of the study. Body weight change (≥10% loss from baseline = humane endpoint) was compared to the control group. Core vital signs monitored at least twice daily included changes in weight, food and fluid intake, activity level, mental status, and respiratory condition. Animals exhibiting any humane endpoint criteria were immediately euthanized according to ARRIVE guidelines. In this study, all experimental animals were euthanized according to the predetermined protocol at the end of the experiment. No unexpected adverse events occurred during the study.

### 2.3. Evaluation of Allergy Symptoms

The two hallmark allergic symptoms (nose rubbing and sneezing) were recorded for 15 min immediately following the final OVA aerosol challenge on day 23, under blinded conditions, as previously described [11].

### 2.4. Cell Counts for Bronchoalveolar Lavage Fluid (BALF)

Following euthanasia, bronchoalveolar lavage fluid (BALF) was collected by instilling and retrieving 1 mL of phosphate-buffered saline (PBS) containing 10% fetal bovine serum (FBS) via a tracheal cannula. After centrifugation, the supernatant was aliquoted and stored at −80 °C, and the cell pellet was resuspended in PBS for total and differential cell counting.

### 2.5. Enzyme-Linked Immunosorbent Assay (ELISA)

Twenty-four hours after the last OVA challenge, the rats were anesthetized and blood samples were collected under sterile conditions via cardiac puncture. Serum was allowed to clot at 4 °C for 1 h and then centrifuged at 1000× *g* for 10 min at room temperature (RT) to separate the serum. The level of OVA-specific IgE in serum was quantitatively determined using an ELISA kit according to the manufacturer’s instructions (Ruixinbio, Quanzhou, China). Absorbance was measured at 450 nm. The concentrations of IL-4, IL-17, interferon (IFN)-γ, and TGF-β in BALF and serum, prepared as described above, were determined using validated ELISA kits (Cloud-Clone, Wuhan, China; Ruixinbio, Quanzhou, China) according to the manufacturers’ instructions.

### 2.6. Histological Assay

On day 23, following euthanasia, the left lung lobes were excised and fixed in 4% paraformaldehyde for 2 weeks and then embedded in paraffin and cut into 5 μm sections. All sections were stained with hematoxylin and eosin (H&E) and evaluated histopathologically for airway and parenchymal inflammation, as well as structural changes, by two independent, blinded observers using a microscope (BX50; Olympus, Tokyo, Japan) equipped with a digital camera (DFC 320; Leica, Wetzlar, Germany).

### 2.7. Microbial Community Analysis

Fresh fecal samples were aseptically collected from rats into sterile 2 mL microcentrifuge tubes and immediately stored at −80 °C. Genomic DNA was extracted from the fecal samples, and the V3–V4 hypervariable regions of the 16S rRNA gene were amplified and sequenced on the Illumina NovaSeq platform by Novogene Co., Ltd. (Beijing, China). The PCR-amplified amplicons were purified using a commercial gel extraction kit according to the manufacturer’s instructions (Qiagen, Hilden, Germany). After quantification, the amplicons were normalized to generate a sequencing library and sequenced using the Illumina platform (Illumina, San Diego, CA, USA). Alpha diversity was determined using Quantitative Insights Into Microbial Ecology (QIIME) 1.7.01. Beta diversity was ascertained using non-metric multi-dimensional scaling (NMDS) in QIIME 1.7.0 to confirm the similarity of species diversity. Significant differences in the relative abundances of bacterial genera among experimental groups were identified via linear discriminant analysis (LDA) effect size (LEfSe).

### 2.8. Statistical Analysis

Data were analyzed using GraphPad Prism 8.0 (GraphPad Software, San Diego, CA, USA). Continuous variables with normal distribution were presented as mean ± standard error of the mean (SEM). Group comparisons were performed using two-tailed, unpaired student’s *t*-test for normally distributed data. Pearson’s correlation coefficients were calculated to assess linear associations between relative abundances of intestinal microbial taxa and cytokine concentrations. Statistical significance was considered as *p* < 0.05.

## 3. Results

### 3.1. Preparation of Probiotics

**Figure 2:**
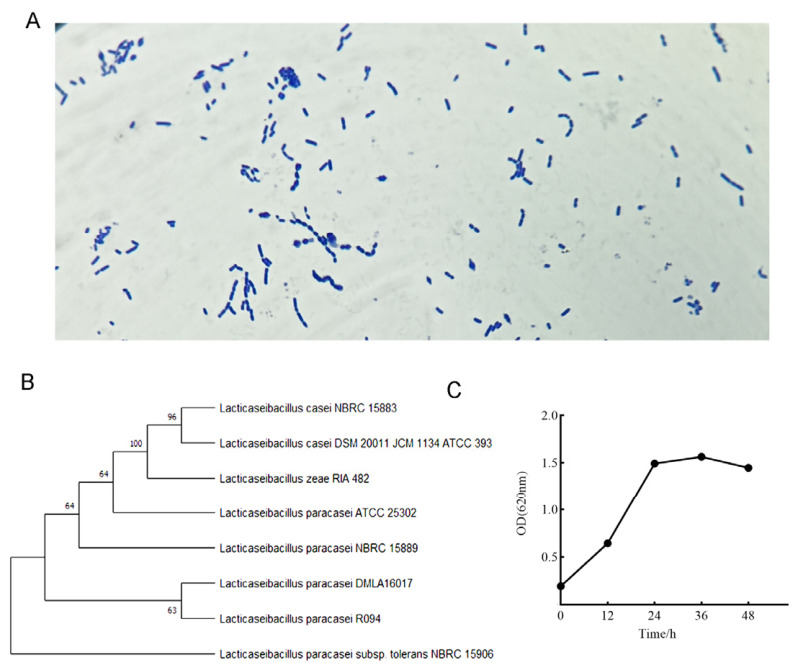
Preparation of DMLA16017. (**A**) Gram-positive bacterium through morphological observation. (**B**) Phylogenetic tree of *Lactobacillus paracasei* strain DMLA16017. (**C**) Growth curve of DMLA16017.

*Lactobacillus paracasei* DMLA16017 was identified as a Gram-positive bacterium with the genetic features and typical morphology of *Lactobacillus* via 16S rDNA sequence analysis. The results are presented in Figure 2. A phylogenetic tree was constructed using MEGA11 software based on homologous sequences from closely related strains, revealing a sequence similarity of up to 99.93% between strain DMLA16017 and *Lacticaseibacillus paracasei* strain R094. The growth curve of DMLA16017 exhibited a typical S-shaped curve, entering the late logarithmic phase and early stationary phase after 24 h of incubation. To ensure stable physiological status and consistent biomass for subsequent experiments, a culture time of 18 h was selected as the sampling point for fermentation, at which time the bacterial concentration reached 1 × 10^9^ cfu/mL.

### 3.2. The VD Influences the Effect of L. paracasei DMLA16017 on Allergic Symptoms

**Figure 3:**
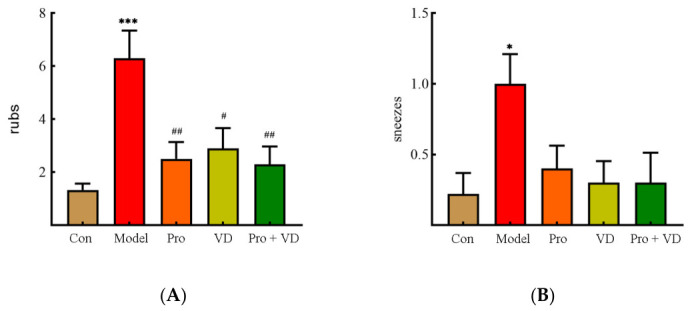
Allergy symptoms resulting from different diets influencing the effect of VD and DMLA16017. (**A**) Number of nose rubs. (**B**) Number of sneezes. *** denotes statistical significance versus the Con group (*p* < 0.001); ## denotes significance versus the AA Model group (*p* < 0.01); # denotes significance versus the AA Model group (*p* < 0.05); * denotes statistical significance versus the Con group (*p* < 0.05).

In this study, allergic symptoms were assessed following OVA challenge. The frequencies of nasal rubbing and sneezing were recorded for each animal over a 15 min observation period. As shown in Figure 3, compared to the control group, the AA model group exhibited significantly higher frequencies of sneezing and nose rubbing (*p* < 0.05, *p* < 0.001). Following administration of DMLA16017, the frequency of nose rubbing was significantly reduced relative to the AA model group (*p* < 0.01). Furthermore, the nose rubbing frequency was significantly decreased in the VD group (*p* < 0.05), with the most pronounced effect observed in the combined intervention group (*p* < 0.01). These findings indicate that the combined intervention of VD and DMLA16017 may exert a synergistic effect in alleviating allergic symptoms.

To investigate the influence of VD intervention on the modulation of allergic responses by DMLA16017, serum levels of OVA-IgE were measured, as shown in Table 1. The results revealed that OVA-IgE levels in the AA model group were significantly elevated compared to those in the control group. In comparison to the AA model group, the combined intervention group demonstrated a more pronounced reduction relative to the DMLA16017 and VD groups individually.

Twenty-four hours after the final OVA challenge, peripheral blood and BALF were collected, and the population of inflammatory cells was quantified (Table 2 and Table 3). Compared to the control group, the AA model group exhibited significant increases in total white blood cell (WBC) counts in the peripheral blood and BALF. Relative to the AA model group, the DMLA16017, VD, and combined intervention groups showed significantly reduced WBC counts in the BALF, with statistically significant differences observed (*p* < 0.05, *p* < 0.05, *p* < 0.01). Furthermore, the combined intervention group demonstrated a more pronounced reduction in WBC count compared to the DMLA16017 and VD groups individually, indicating a trend toward enhanced efficacy. These findings suggest that VD and DMLA16017 can modulate immune cell populations in the BALF and effectively suppress white blood cell infiltration, thereby alleviating airway inflammation.

### 3.3. Airway Inflammation in AA Rats with Co-Administration of VD and DMLA16017

**Figure 4:**
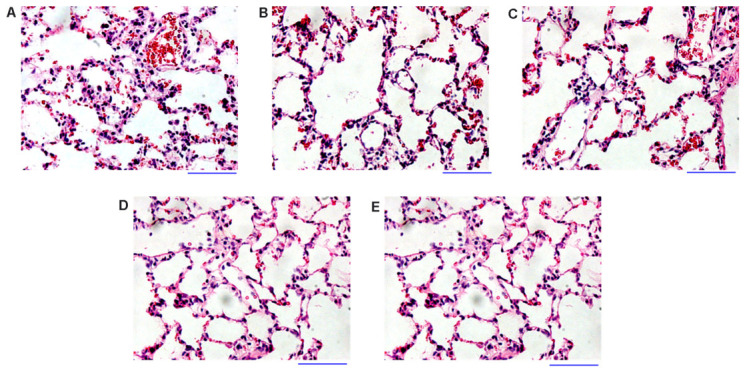
Morphological evaluation of lung tissue. (**A**) AA Model Group. (**B**) DMLA16017 Group. (**C**) VD Group. (**D**) Combined intervention of VD and DMLA16017 Group. (**E**) Control Group.

Histological analysis was performed on the lung tissues to evaluate pathological changes associated with airway inflammation. Following H&E staining, the eosinophil cytoplasm appeared red (Figure 4). Compared to the control group, the AA model group exhibited the typical histopathological features of AA, including marked structural disruption, thickened alveolar septa, and alveolar destruction with evidence of fusion. In comparison to the AA model group, the DMLA16017, VD, and combined intervention groups showed reduced eosinophil infiltration. Furthermore, the combined intervention group displayed less inflammatory cell infiltration, better-preserved alveolar architecture, and reduced alveolar fusion compared to the DMLA16017 and VD groups alone. These findings indicate that both VD and DMLA16017 interventions can attenuate lung tissue injury, with combination therapy demonstrating enhanced efficacy in suppressing airway inflammation.

### 3.4. Alteration of Cytokine Levels by Combined Intervention of VD and DMLA16017

Following OVA challenge, this study measured cytokine levels in the BALF and serum to further investigate the effects of various intervention strategies on the balance of Th1/Th2 and Th17/Treg immune responses and to elucidate the underlying immunomodulatory mechanisms of VD in combination with DMLA16017. As illustrated in Table 4, Table 5, Table 6 and Table 7, the immunomodulatory effects of DMLA16017 on the AA model varied significantly under different dietary conditions. Compared to the AA model group, IL-4 levels in the DMLA16017, VD, and combined intervention groups exhibited a decreasing trend (*p* = 0.0702) in Table 4 and Table 5. Moreover, IFN-γ levels in the combined intervention group were higher than those in the DMLA16017 and VD groups, with a statistically significant difference (*p* < 0.05) (Table 4 and Table 5). Additionally, the level of IL-17, a Th17-associated cytokine, was significantly reduced in the combined intervention group relative to that in the AA model group (*p* < 0.05) in Table 6 and Table 7. In comparison to the Con group, the AA model group showed a marked reduction in the TGF-β level (*p* < 0.05), whereas the DMLA16017, VD, and combined intervention groups all demonstrated a tendency toward increased TGF-β levels compared to the AA model group (Table 6 and Table 7). These findings suggest that the combined administration of VD and DMLA16017 effectively suppresses the expression of Th2- and Th17-related cytokines in both lung tissue and serum while enhancing the production of Th1 and Treg related cytokines, thereby exerting a synergistic effect in restoring immune homeostasis and attenuating allergic inflammatory responses.

### 3.5. Profiling Bacterial Sequences

**Figure 5:**
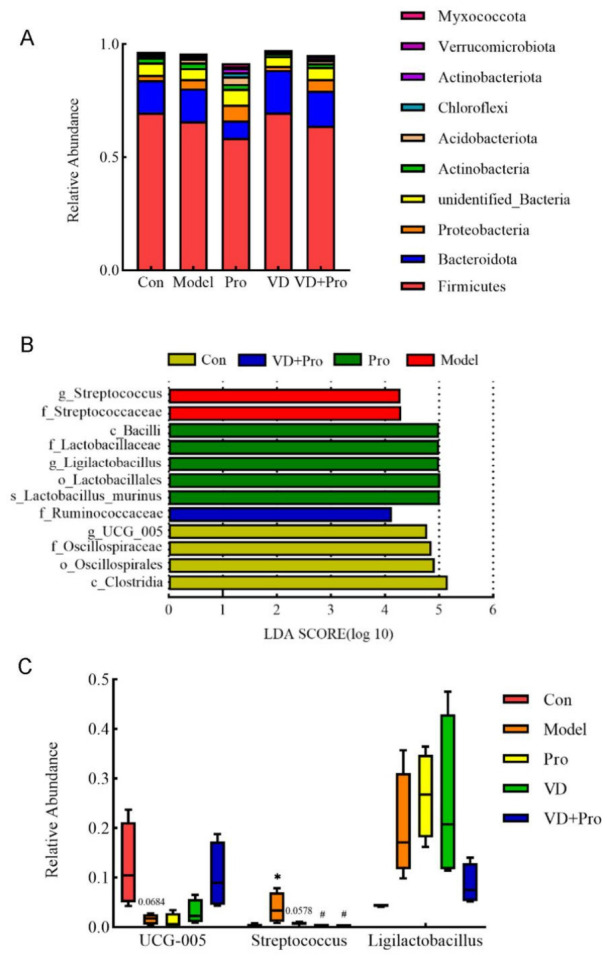
Gut microbiota comparison analysis. (**A**) Phylum level of intestinal microbiota composition. (**B**) LEfSe analysis. (**C**) The relative abundances of three dominant bacterial strains. * indicates a significant difference compared to the Con group, *p* < 0.05. ^#^ indicates a significant difference compared to the AA Model group (*p* < 0.05).

In order to understand the species composition of the community at various levels and effectively determine the composition of the community structure, 16S RNA sequencing was performed on respiratory samples. We selected operational taxonomic units with a relative abundance greater than 2% of total sequence reads to generate a species classification display chart, as shown in Figure 5.

At the phylum level of intestinal microbiota composition in rats, Firmicutes and Bacteroidetes exhibited the highest relative abundances (Figure 5A). Compared to the control group, the AA model group showed a significant increase in the relative abundance of Firmicutes and a significant decrease in that of Bacteroidetes. UCG-005 was predominant in the control group, Ligilactobacillus dominated in the DMLA16017 group, and Streptococcus was the dominant genus in the AA model group. Relative to the control group, the AA model group displayed a reduced relative abundance of UCG-005 and an increased abundance of Streptococcus (*p* = 0.0684; *p* < 0.05). In comparison to the AA model group, the DMLA16017 group and the VD group exhibited an increased relative abundance of Ligilactobacillus. Additionally, in both the VD group and the VD combined with DMLA16017 group, the relative abundance of UCG-005 increased, while the abundance of Streptococcus significantly decreased (*p* < 0.05).

Based on the LEfSe analysis (Figure 5B,C), at the genus level of intestinal microbiota composition across experimental groups, UCG-005 was identified as the dominant taxon in the control group, Ligilactobacillus predominated in the DMLA16017 group, and Streptococcus emerged as the dominant genus in the AA model group, with all exhibiting significantly higher relative abundances compared to the other groups (*p* < 0.05). Notably, the relative abundance of UCG-005 in the combined intervention group was closer to that observed in the control group. These findings indicate that inflammatory responses can markedly alter the structural composition of the gut microbiota at both the phylum and genus levels, whereas interventions with VD and DMLA16017 confer partial protective and restorative effects, with the combination therapy demonstrating a more pronounced beneficial impact.

**Figure 6:**
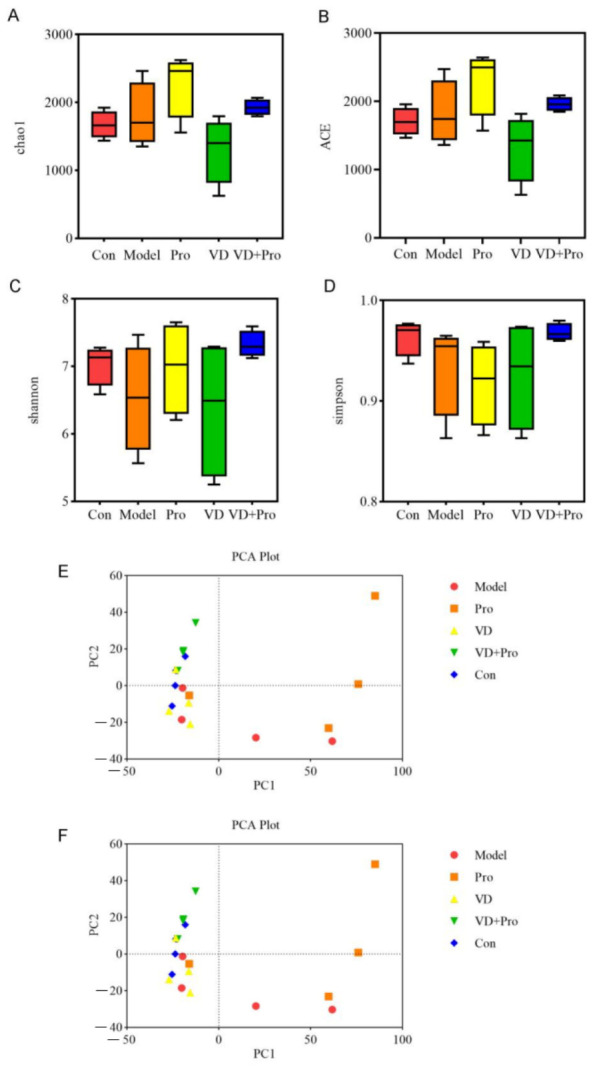
Diversity in microbiota community structure in different rat groups. (**A**) Chao1 index. (**B**) ACE index. (**C**) Shannon index. (**D**) Simpson index. (**E**) PCA plots. (**F**) PCoA plots.

To detect alterations in species abundance and diversity in each group, we analyzed the diversity of the bacterial communities. In this study, alpha diversity indices, including Shannon (community evenness), Simpson (community diversity), ACE (community richness), and Chao1 (community richness), were assessed. For comparisons among more than two groups, the Kruskal–Wallis test was employed. As shown in Figure 6, compared to the AA model group, the DMLA16017 group and the VD combined with DMLA16017 group exhibited increased Chao1 and ACE index values (Figure 6A,B), indicating a trend toward higher microbial richness. These results suggest that inflammatory responses can alter the compositional diversity of the gut microbiota, while VD and DMLA16017 exert regulatory effects. Analysis of the Shannon and Simpson indices further revealed that inflammation impacts both the abundance and evenness of intestinal microbial communities (Figure 6D,E). Specifically, the AA model group showed significantly lower Shannon and Simpson indices compared to the control group, indicating reduced microbial diversity. By contrast, treatment with DMLA16017 or VD partially restored these indices, with the combination therapy demonstrating the most pronounced ameliorative effect.

In the beta diversity analysis, greater similarity in community composition between samples corresponds to shorter distances in PCA (principal component analysis) and PCoA (principal coordinate analysis) plots (Figure 6E,F). Samples from different treatment groups typically exhibit distinct clustering patterns, reflecting specific microbial profiles. The control group, the VD group, and the VD combined with DMLA16017 group displayed tight and well-defined spatial clustering, whereas the AA model group and the DMLA16017 group showed relatively dispersed distributions. This indicates lower variation in microbial composition within the VD intervention and combination therapy groups. Notably, the microbial community structure of the combination treatment group was most similar to that of the normal control group. Principal coordinate analysis (PCoA) further supported the protective effect of combined VD and DMLA16017 administration on the intestinal microbiota.

### 3.6. Correlation Analysis of the Intestinal Microbiota and Cytokine Expression in BALF

To further investigate the association between predominant intestinal microbiota and cytokines in the BALF, Pearson’s correlation analysis was performed in this study. As shown in Table 8, Streptococcus exhibited a significant positive correlation with IL-4 (r = 0.9681, *p* = 0.0068) and IL-17 (r = 0.9542, *p* = 0.0117) and a significant negative correlation with IFN-γ (r = −0.9785, *p* = 0.0038). These findings suggest that increased abundance of Streptococcus may promote inflammatory responses, indicating its potential involvement in the pathogenesis of AA.

## 4. Discussion

Allergic disorders are a major global public health concern, and common manifestations include asthma, allergic rhinitis, atopic dermatitis, and food allergies [12]. Among these conditions, asthma substantially compromises a patient’s life quality, may be life-threatening during acute exacerbation, and imposes substantial medical and socioeconomic burdens. Because clinical management of asthma primarily relies on drug interventions, there is an urgent need to identify and develop novel probiotic strains for the prevention and treatment of allergic disorders. In this study, a rat model of AA was established to systematically investigate the effects of combined intervention with VD and *L. paracasei* DMLA16017 on physiological parameters, pathological features, immune regulatory mechanisms, and the gut microbiota. The findings provide a robust theoretical foundation for the development of next-generation probiotics and personalized therapies.

Interactions among VD, the gut microbiome, and immune system can occur at several levels and may include the innate and the adaptive immune system. This study involved the taxonomic identification of *L. paracasei* DMLA16017 and fermentation process to determine the optimal culture duration and conditions. Utilizing the AA model, biomarkers related to inflammation and the immune response were measured in lung tissue, serum, BALF, and fecal samples. The results demonstrated that OVA induction leads to significant pathological alterations in lung tissue. Furthermore, there was marked dysbiosis of the gut microbiota, characterized by reduced species richness and altered composition of microbial communities. Combined intervention with VD and *L. paracasei* DMLA16017 effectively ameliorated physiological symptoms and pulmonary histopathological damage and enhanced microbial diversity. These findings suggest that the synergistic effects of VD and *L. paracasei* DMLA16017 may provide the foundation for the development of novel dietary interventions and therapeutic strategies.

There is a growing awareness that the composition of the gut microbiome is linked with respiratory disease on the gut–lung axis [13]. Significant cross-talk exists between the immune systems of the gut and lung, whereby the intestinal microbiota may contribute to mucosal integrity through the fermentation of dietary fiber to produce SCFAs, which exert protective effects on the mucus layer, support intestinal microecological balance, and directly modulate pulmonary immune responses to prime host defenses against invading pathogens [14]. In the present study, the species abundance and evenness of the gut microbiota were significantly higher in the combined intervention group (VD and *L. paracasei* DMLA16017) compared with those in the AA model group, which indicates that the combined treatment enhances microbial diversity and strengthens the host’s capacity to resist inflammatory responses. This supports the stabilizing and beneficial impact of this intervention on intestinal microecological homeostasis.

Asthma may lead to structural alterations in the respiratory tract. It induces airway injury, accompanied by persistent and intensified inflammation, which results in airway remodeling. Airway fibrosis represents a key histopathological hallmark of chronic inflammatory repair and is primarily driven by excessive deposition of extracellular matrix (ECM) mediated by activated airway fibroblasts [15]. While this fibrotic response may offer partial protection to airway epithelial cells against inflammation, it promotes narrowing of the bronchial lumen and impairs ventilatory function. Accumulating evidence indicates that intervention using VD suppresses activation of the transforming growth factor-beta (TGF-β)/Smad signaling pathway and downregulates the expression of miR-21, which leads to significant reductions in the expression of type I collagen, α-smooth muscle actin (α-SMA), and fibronectin in TGF-β1-stimulated human bronchial fibroblasts (HBFs) [16]. Collectively, these effects help inhibit aberrant accumulation of ECM and attenuation of airway remodeling. Experimental findings revealed statistically significant elevations in white cell counts in the BALF and peripheral blood in the AA model group compared to those in the control group. By contrast, the *L. paracasei* DMLA16017 group, VD group, and the combination intervention group exhibited reduced levels of leukocytes relative to those in the AA model group, with the greatest reduction observed in the combination group. These results suggest that co-administration of VD and *L. paracasei* DMLA16017 exerts a potent anti-inflammatory effect in rats, which effectively mitigates systemic and pulmonary inflammation.

A study using a mouse model of AA demonstrated a significantly negative correlation between serum levels of 25(OH)D and the mass of airway smooth muscle (ASM) [17]. Increased intake of VD suppresses thickening of the ASM, airway subepithelial fibrosis, and excessive goblet cell hyperplasia. In the AA model group, histopathological examination of the lung tissue revealed marked inflammatory cell infiltration (predominantly eosinophils), along with substantial accumulation of eosinophils in the bronchial lumen, thickened alveolar septa, and structural damage characterized by alveolar destruction and fusion. By contrast, the *L. paracasei* DMLA16017 group, VD group, and combined intervention group exhibited reduced eosinophilic infiltration. Furthermore, the VD and *L. paracasei* DMLA16017 groups displayed the least extent of inflammatory cell infiltration, the most preserved alveolar architecture, and minimal alveolar fusion. Collectively, these findings suggest that the administration of VD and *L. paracasei* DMLA16017 can mitigate pulmonary damage in AA, and the combination therapy demonstrates superior protective efficacy.

The pathogenesis of AA is highly complex. Naive CD4+ Th0 cells can differentiate into Th1 or Th2 lineages under the influence of distinct cytokine and chemokine microenvironments [18]. Upon exposure to allergens, dendritic cells (DCs)—specialized antigen-presenting cells (APCs)—recognize, internalize, and process antigens, which results in the presentation of antigenic peptide fragments to naive CD4+ T lymphocytes via major histocompatibility complex (MHC) class II molecules. Allergen re-exposure triggers rapid degranulation, which results in the release of a wide array of inflammatory mediators (such as histamine, leukotrienes, and proteases) that collectively induce airway hyperresponsiveness [19]. Chronic or intensified inflammation may lead to further structural changes of airways. Moreover, immune dysregulation involving the imbalance between regulatory T cells (Tregs) and Th17 cells plays a critical role in the progression of asthma [20,21]. Activation of the interleukin (IL)-17 signaling pathway is implicated in the amplification of allergen-induced airway inflammation. Imbalance of the Treg/Th17 ratio may contribute to impaired immune tolerance, while the ratio of IL-4/interferon gamma (IFN-γ) serves as a reliable indicator of Th1/Th2 immune balance [22]. This study demonstrated that combined intervention with VD and *L. paracasei* DMLA16017 was associated with increased levels of IFN-γ, reduced expression of Th2-related cytokines in lung tissue, and enhanced production of Th1-related cytokines. These findings suggest that the combined intervention causes a shift toward Th1 polarization for mucosal immunity. Collectively, combined intervention effectively modulates the pulmonary Th1/Th2 immune response and ameliorates the state of immune dysregulation.

*Lacticaseibacillus paracasei* is a member of the *Lactobacillus casei* group within the family *Lactobacillaceae* and is commonly present in fermented dairy products and the gastrointestinal tract [23]. *L. paracasei* can effectively modulate airway inflammation associated with AA through the activation of Th1 cell-mediated immune responses and promotion of the differentiation and expansion of Tregs [24]. Combined intervention significantly reduced systemic levels of IL-17, downregulated the expression of Th17-related cytokines in lung tissue, and upregulated Treg-associated production of cytokines, which synergistically restored the pulmonary Treg/Th17 immune balance. In this study, a newly isolated strain (*L. paracasei* DMLA16017) was administered in combination with VD to a rat model of AA. Compared to the AA model group, the VD intervention group exhibited markedly attenuated lung tissue pathology, reduced inflammatory cell infiltration, and improved bronchial lumen stenosis, which substantiates the protective effects of the combined intervention against pulmonary injury.

Immunoglobulin E (IgE) is an evolutionarily conserved component of the innate immune defense system, and allergic reactions are typically characterized by elevated levels of IgE [25]. Upon re-exposure to the same allergen, crosslinking of surface-bound IgE triggers rapid degranulation, which results in the release of a wide array of inflammatory mediators that initiate immediate-type hypersensitivity reactions [26]. These findings are consistent with this mechanism because combined intervention positively modulated the levels of IgE and mitigated inflammatory responses through the reduction of circulating concentrations of IgE. Therefore, probiotics may ameliorate AA through modulation of the intestinal microbiota, and their efficacy is potentially influenced by environmental factors such as dietary composition. Supplementation with *L. paracasei* DMLA16017 increased levels of TGF-β and IFN-γ in the serum and BALF in rats, while levels of IL-4 and IL-17 exhibited a significant downward trend. These results closely align with existing evidence and support the conclusion that the combination of VD and DMLA16017 effectively attenuates the pathological progression of allergic asthma through targeted regulation of immune response mechanisms.

Vitamin D plays a crucial and multifaceted role in the regulation of the immune system. It modulates the activity of T lymphocytes, suppresses pro-inflammatory immune responses, mitigates the onset and progression of autoimmune disorders, and contributes to the maintenance or restoration of immunological homeostasis between Th1/Th2 and Th17/Treg immune responses. Specifically, VD inhibits the differentiation and maturation of Th17 cells and suppresses the production of IL-17 through downregulation of the expression of nuclear factor of activated T cells (NFAT) and the transcription factor RUNX1 [27]. Conversely, it promotes the expression of forkhead box P3 (FOXP3) in naive CD4+ T cells, which facilitates the development and functional specialization of Tregs [28]. Mature Tregs exert anti-inflammatory effects through the secretion of IL-10 and TGF-β, interaction with APCs, and suppression of helper T-cell activation, which ultimately reduces inflammatory responses and protects against the exacerbation of asthma symptoms. Allergic asthma frequently exhibits reduced numbers and impaired function of Tregs. Supplementation with VD significantly upregulated the expression of immune checkpoint molecules CTLA-4 and PD-1 in Tregs, increased the population of Tregs, and enhanced systemic immune tolerance. Vitamin D effectively modulates immune function, attenuates airway inflammation, lowers the risk of airway remodeling, and controls the progression of AA [29]. In this study, Pearson’s correlation analysis was employed to assess the associations between gut microbiota composition and cytokine profiles. The abundance of *Streptococcus* positively correlated with the levels of IL-4 and IL-17 and negatively correlated with the levels of IFN-γ, which support the notion that enrichment with *Streptococcus* may promote a pro-inflammatory state, implicating this genus in the pathogenesis of allergic asthma. These findings suggest that combined intervention with VD and *L. paracasei* DMLA16017 may alleviate inflammation by modulating or suppressing proliferation of the gut microbiome, altering the production of microbial metabolites, and influencing host immune responses.

This study has several limitations. First, AA exhibits a complex, multifactorial pathogenesis; thus, more physiologically relevant germ-free animal models or in vitro models are required to rigorously assess the synergistic effects on combined intervention with VD and *L. paracasei* DMLA16017. Second, current methodologies for identifying and isolating functionally critical regulatory fatty acids remain limited. Future studies should employ targeted metabolomic profiling to pinpoint specific fatty acid species with demonstrable anti-infective and anti-inflammatory properties, thereby strengthening mechanistic understanding and informing rationally designed therapeutic interventions.

## 5. Conclusions

In this research, the therapeutic effects of the co-administration of *L. paracasei* DMLA16017 with VD in rats with AA was systematically investigated. The evaluation encompassed physiological status, pathological features, immune regulatory mechanisms, and composition of the gut microbiota. This research provides a robust theoretical foundation and scientific evidence for the development of novel biological therapeutics and personalized clinical treatment strategies.

## Figures and Tables

**Table 1:** Content of OVA-IgE in serum of rats ($\bar{x}\pm s$).

Group	n	IgE (ng/mL)
Con	6	216.57 ± 10.90
Model	6	303.61 ± 26.43 *
Pro	6	243.88 ± 16.09
VD	6	271.40 ± 17.40
VD+ Pro	6	224.64 ± 27.28

* indicates a significant difference compared to the AA Model group (*p* < 0.05).

**Table 2:** White blood cell count in peripheral blood of rats ($\bar{x}\pm s$).

Group	n	WBC (10^6^/L)
Con	6	4.23 ± 0.68
Model	6	7.53 ± 1.12 *
Pro	6	5.81 ± 0.40
VD	6	5.40 ± 0.48
VD+ Pro	6	4.84 ± 0.36

* indicates a significant difference compared to the Con group (*p* < 0.05).

**Table 3:** White blood cell count in BALF of rats ($\bar{x}\pm s$).

Group	n	WBC (10^6^/L)
Con	6	71.23 ± 6.27
Model	6	126.62 ± 14.18 **
Pro	6	84.27 ± 7.00 ^#^
VD	6	80.38 ± 9.93 ^#^
VD+ Pro	6	76.43 ± 8.94 ^##^

** indicates a significant difference compared to the Con group (*p* < 0.001); ^##^ indicates a significant difference compared to the AA Model group (*p* < 0.01); ^#^ indicates a significant difference compared to the AA Model group (*p* < 0.01).

**Table 4:** Contents of IL-4 and IFN-γ in BALF of rats ($\bar{x}\pm s$).

Group	n	IL-4 (pg/mL)	IFN-γ (pg/mL)
Con	6	43.01 ± 3.33	257.45 ± 16.02
Model	6	55.69 ± 2.78 *	200.54 ± 13.79
Pro	6	48.28 ± 3.07	239.94 ± 10.40
VD	6	45.04 ± 2.91	255.94 ± 11.90
VD+ Pro	6	44.23 ± 2.41	266.24 ± 26.63

* indicates a significant difference compared to the Con group, *p* < 0.05.

**Table 5:** Contents of IL-4 and IFN-γ in serum of rats ($\bar{x}\pm s$).

Group	n	IL-4 (pg/mL)	IFN-γ (pg/mL)
Con	6	84.91 ± 6.93	266.80 ± 15.34
Model	6	134.95 ± 9.05 ***	207.10 ± 21.26
Pro	6	115.92 ± 4.29	256.02 ± 13.36
VD	6	106.05 ± 10.82	267.70 ± 12.73
VD+ Pro	6	91.51 ± 4.60 ^##^	273.12 ± 8.55 ^#^

*** indicates a significant difference compared to the Con group (*p* < 0.001). ^##^ indicates a significant difference compared to the AA Model group (*p* < 0.01). ^#^ indicates a significant difference compared to the AA Model group (*p* < 0.05).

**Table 6:** Contents of TGF-β and IL-17 in BALF of rats ($\bar{x}\pm s$).

Group	n	TGF-β (pg/mL)	IL-17 (pg/mL)
Con	6	49.46 ± 3.10	55.71 ± 3.39
Model	6	41.91 ± 2.10	74.88 ± 4.51
Pro	6	44.29 ± 1.98	62.13 ± 9.64
VD	6	44.52 ± 2.62	62.56 ± 4.91
VD+ Pro	6	45.92 ± 2.21	57.61 ± 3.72

**Table 7:** Contents of TGF-β and IL-17 in serum of rats ($\bar{x}\pm s$).

Group	n	TGF-β (pg/mL)	IL-17 (pg/mL)
Con	6	31.40 ± 2.69	87.95 ± 5.04
Model	6	23.82 ± 1.36 *	104.14 ± 6.59
Pro	6	26.99 ± 1.45	93.62 ± 4.57
VD	6	26.64 ± 1.03	93.46 ± 5.67
VD+ Pro	6	26.30 ± 1.63	88.55 ± 4.69

* indicates a significant difference compared to the Con group, *p* < 0.05.

**Table 8:** Analysis of the relationship between intestinal flora and cytokines.

Gut Flora	IL-4		IL-17		IFN-γ		TGF-β	
	r	*p*	r	*p*	r	*p*	r	*p*
Ligilactobacillus	0.4712	0.4231	0.5258	0.3629	−0.4114	0.4914	−0.7569	0.5730
Lactobacillus	−0.2676	0.6630	−0.1822	0.7690	0.1182	0.8500	0.6022	0.2820
Streptococcus	0.9681	0.0068 **	0.9542	0.0117 *	−0.9785	0.0038 **	−0.7164	0.1734
Turicibacter	−0.4392	0.4594	−0.2004	0.7466	0.3918	0.5142	0.2203	0.7184
Ruminococcus	−0.7458	0.1478	−0.6270	0.2576	0.7562	0.1391	0.6042	0.2805
Limosilactobacillus	−0.5581	0.3282	−0.5499	0.3369	0.5625	0.3236	0.2471	0.6887
UCG-005	−0.7033	0.1851	−0.7351	0.1570	0.6507	0.2344	0.8757	0.0516
Romboutsia	−0.6484	0.2366	−0.5545	0.3320	0.5177	0.3716	0.8268	0.0842
Candidatus_Saccharimonas	−0.5202	0.3688	−0.3755	0.5334	0.4351	0.4640	0.6149	0.2697
Staphylococcus	−0.4514	0.4454	−0.4952	0.3963	0.2738	0.6558	0.8490	0.0688

** indicates *p* < 0.01; * indicates *p* < 0.05.

## Data Availability

The original contributions presented in the study are included in the article. Further inquiries can be directed to the corresponding author.

## Abbreviations

α-SMA	Alpha-smooth muscle actin
AA	Allergic asthma
APC	Antigen-presenting cells
ASM	Airway smooth muscle
BALF	Bronchoalveolar lavage fluid
DC	Dendritic cell
ECM	Extracellular matrix
FOXP3	Forkhead box P3
HBF	Human bronchial fibroblasts
IFN-γ	Interferon gamma
IgE	Immunoglobulin E
IL	Interleukin
MHC	Major histocompatibility complex
NFAT	Nuclear factor of activated T cells
OVA	Ovalbumin
SCFA	Short-chain fatty acid
TGF-β	Transforming growth factor-beta
Th1	T-helper 1
Th2	T-helper 2
Tregs	Regulatory T cells
VD	Vitamin D
